# %dd-cfDNA: The New Frontier for Heart/Lung Transplant Surveillance?

**DOI:** 10.3389/ti.2025.15555

**Published:** 2025-12-29

**Authors:** Sean Agbor-Enoh, Ethan Fraser, Nitin Nadella, Temesgen E. Andargie, Muhtadi Alnababteh

**Affiliations:** 1 Genomic Research Alliance for Transplantation (GRAfT), Bethesda, MD, United States; 2 Laboratory of Applied Precision Omics, National Heart, Lung, and Blood Institute (NHLBI), NIH, Bethesda, MD, United States; 3 Department of Medicine, School of Medicine, Johns Hopkins University, Baltimore, MD, United States; 4 Critical Care Medicine Department (CCMD), NIH, Bethesda, MD, United States; 5 University of Missouri-Kansas City School of Medicine, University of Missouri, Kansas City, Kansas City, MO, United States; 6 Department of Medicine, University of Maryland Medical Center, Baltimore, MD, United States

**Keywords:** cfDNA, acute rejection, diagnosis, transplantation, allograft injury

## Abstract

Transplantation improves survival and quality of life, but rejection remains a major threat to allograft longevity. Current surveillance relies heavily on protocols with clinically indicated biopsies, which are invasive, carry procedure-related risks, and have variable sensitivity due to sampling and interpretation limitations. Percent donor-derived cell-free DNA (%dd-cfDNA) has emerged as a noninvasive blood-based biomarker for allograft injury and a potential rule-out test for rejection. Centralized commercial assays are increasingly used in clinical practice; however, published studies report heterogeneous performance and reveal important blind spots and confounders. This review synthesizes the evidence for %dd-cfDNA in thoracic transplantation, delineates its limitations, and outlines emerging cfDNA methodologies that may reduce reliance on invasive biopsies and enable more individualized monitoring strategies.

## Introduction

Acute rejection (AR) remains a critical vulnerability in thoracic transplantation. Clinicians rely on traditional biopsies of the allograft to detect AR and two classic phenotypes: acute cellular rejection (ACR) and antibody-mediated rejection (AMR). The traditional one-size-fits-all monitoring protocol performs repeated surveillance biopsies to detect and treat early forms of AR before irreversible allograft injury, chronic rejection, and allograft failure develop. Testing often necessitates intricate coordination among various specialties, procedural services, and advanced care planners [1, 2]. This complex model places a substantial burden on healthcare systems and patients alike, ultimately imposing significant socioeconomic across a wide spectrum of care [1, 3, 4]. Moreover, the low sensitivity and high inter-rater variability of biopsy further compromise transplant outcomes [5, 6].

In light of these challenges, donor-derived cell-free DNA (dd-cfDNA) has emerged as a highly sensitive and non-invasive alternative to biopsy. Cell-free nucleic acids are circulating DNA and RNA fragments (cfDNA and cfRNA, respectively) that are released from nuclear, mitochondrial, or microbial genomes into the peripheral bloodstream at the time of cell injury and/or death. In transplant patients, both the recipient and donor contribute to the circulating cell-free nucleic acid pool (rd-cfDNA and dd-cfDNA, respectively).

Cohort studies among transplant patients demonstrate excellent diagnostic performance of dd-cfDNA, with high negative predictive values when used to screen for acute rejection, primary graft dysfunction, and chronic rejection in clinically stable patients [7]. With the availability of commercial testing, %dd-cfDNA has been increasingly adopted in routine clinical care at US and European Centers, particularly large academic institutions [8–10]. Clinical experiences, however, have been mixed: some centers report consistent performance aligning with early cohort findings, while others exhibit less favorable results alongside challenges with interpretation [7, 11, 12]. To optimize integration into routine clinical care, it is paramount to address the blind spots of %dd-cfDNA and move beyond the one-size-fits-all monitoring paradigm in thoracic transplant populations.

This review aims to highlight the strengths, blind spots, and novel approaches using cfDNA to address these dd-cfDNA gaps.

## Historical Basis: The Advent of Cell-free DNA Technology

**FIGURE 1:**
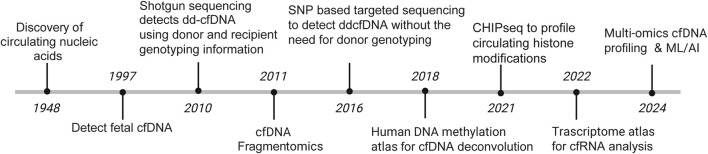
Evolution of cfDNA technologies.

Transplantation restores organ function and creates a donor–recipient genomic admixture wherein measurement of cell-free DNA (cfDNA) provides a noninvasive window into allograft health. Half a century after the discovery of cfDNA in human plasma, Denis Lo first reported on the presence of fetal DNA in maternal plasma in 1997 [13]. Despite these initial findings, cfDNA adoption in transplantation was initially slow. In 2010, Dr. Stephen Quake published a SNP-based approach that leveraged the unique transplant genomic admixture [14]. This 1st generation assay genotyped transplant donors and recipients to identify informative donor-recipient single nucleotide polymorphisms (SNPs). Post-transplant plasma was then subjected to cfDNA isolation and whole genome sequencing and reads were analyzed using SNPs to assign donor and recipient cfDNA fragments. Percent dd-cfDNA (%dd-cfDNA) was then computed as the donor-to-total (donor plus recipient) cfDNA percentage, which has become the standard reported value in transplant populations. Since then, commercial %dd-cfDNA tests are increasingly available for routine clinical care. These SNP-based assays used a targeted approach and imputed donor and recipient SNPs without the need for genotype data. Figure 1 summarizes the modern evolution of cfDNA technologies.

Novel cfDNA approaches (3rd generation) have emerged in the last decade and while they do not fix the listed weaknesses, they offer mechanisms that utilize epigenetic fingerprints on cfDNA to better characterize tissue-specific contributions and highlight molecular mechanisms of action [15]. In theory, these approaches could unveil known and unknown pathobiological information of AMR and ACR using a single vial of blood. We summarize these different epigenetic technologies at the end of this review.

## Current Knowledge

### Cell-Free DNA to Detect Acute Rejection

Table 1 summarizes seminal studies on the use of cfDNA in thoracic transplant, highlighting the wide range of %dd-cfDNA cutoffs used in initial validation studies and the ongoing work that must be done before %dd-cfDNA is widely adopted. The Stanford Genome Transplant Dynamics (GTD) team launched the initial transformative studies in both heart and lung transplants. The NHLBI-funded Genomic Research Alliance for Transplantation (GRAfT) consortium has since built on these initial studies.

**TABLE 1:** Seminal Studies in the Validation of dd-cfDNA as a marker for thoracic organ rejection.

Transplant type	Author	Study design	Sample size (n)	Biomarker threshold	Data Collection methodology	AUROC	Specificity (%)	Sensitivity (%)	PPV (%)	NPV (%)
Heart transplantation	De Vlaminck et al. [16]	Single-center prospective cohort study	65	Dd-cfDNA ≥0.25% to detect acute cellular rejection (ISHLT ≥2R/3A) or AMR	SNP-based shotgun sequencing	0.83	58	93	-	-
	Khush et al. [17]	Multicenter prospective cohort study + single center cohort study	773	ddcfDNA ≥0.2% to detect acute rejection	Allosure	0.64	80	44	8.9	97.1
	Agbor-Enoh et al. [18]	Multicenter prospective cohort study	171	Day 28 ddcfDNA ≥0.25% to detect biopsy-positive acute rejection	SNP-based shotgun sequencing	0.92	81	85	19.6	99.2
	Knuttgen et al. [19]	Single-center prospective cohort study	87	ddcfDNA ≥0.35% to detect severe rejection (ISHLT 1R/2R)	Therasure transplant monitor	0.81	83	76	31	97
	Kim et al. [20]	Observational single-center cohort study	223	ddcfDNA ≥0.15% to detect acute rejection	Prospera	0.86	76.9	78.5	25.1	97.3
				Absolute quantity ddcfDNA ≥13 cp/mL to detect acute rejection		0.88	82.5	84	32.2	98.1
	Bohmer et al. [21]	Multicenter observational prospective cohort study	94 (24 children/70 adults)	Absolute quantity ddcfDNA ≥25 cp/mL to detect biopsy-confirmed rejection	SNP-based shotgun sequencing approach	0.87	80.7	94.1	8.6	99.9
				ddcfDNA ≥0.09% to detect biopsy-confirmed rejection		0.75	49.3	88.2	3.1	99.6
Lung transplantation	De Vlaminck et al. [22]	Single-center prospective cohort study	51	ddcfDNA ≥1.0% to detect moderate-to-severe acute rejection	SNP-based shotgun sequencing	0.9	100	73	-	-
	Jang et al. [23]	Multicenter prospective cohort study	148	Day 45 ddcfDNA ≥0.5% to detect acute rejection	SNP-based shotgun sequencing	0.89	65	95	51	96
				Day 45 ddcfDNA ≥1.0% to detect acute rejection		-	84	77	64	90
	Keller et al. [24]	Multicenter retrospective cohort study	175	ddcfDNA ≥1.0% to detect acute lung allograft dysfunction	SNP-based shotgun sequencing	0.79	70	76.2	66.7	79.2
	Rosenheck et al. [25]	Single-center prospective cohort study	104	Day 45 ddcfDNA ≥1.0% to detect acute rejection	Prospera	0.91	89.1	82.9	51.9	97.3
				Day 45 ddcfDNA ≥1.0% to detect combined allograft injury (ACR + AMR + CLAD/NRAD + INFXN)		0.76	59.9	83.9	-	-
	Ju et al. [26]	Single-center retrospective cohort study	188	Prediction score based upon ddcfDNA and mNGS ≥0.2781 to detect rejection	Allodx (NGS ddcfDNA system based upon analysis of 6200 SNPs)	0.986	94.7	98.2	88.7	99.2

#### Lung Transplant

In 2015, using the 1st generation SNP-based assay to measure %dd-cfDNA, De Vlaminck et al. reported excellent diagnostic performance with a ≥1% dd-cfDNA threshold used as indicator of AR when compared to traditional metrics of AR detection [22]. Lower grade ACR and AMR (A1 and A2) were not included.

Building on this initial data, GRAfT replicated and validated the cfDNA detection methods established by the Stanford GTD, enhancing the reliability and clinical applicability of %dd-cfDNA in assessing AR, which now included AMR and lower grades of ACR [27]. This early work resulted in a seminal publication by Jang et al. who proposed two %dd-cfDNA rejection detection thresholds of 1% and 0.5% dd-cfDNA indicative of a high and low risk patients, respectively [23]. This work set the stage for further clinical testing and validation studies, many of which have proposed different diagnostic thresholds to maximize the sensitivity and specificity of dd-cfDNA [25, 28–31].

In 2022, Keller et al. from the GRAfT consortium reported %dd-cfDNA performance as part of routine clinical care using a home-based surveillance program and thresholds from Jang et al. %dd-cfDNA demonstrated excellent diagnostic performance for detection of acute lung allograft dysfunction (ALAD–defined in this study as a composite endpoint of either acute rejection or infection) and its use successfully avoided 80% of bronchoscopies, which aligned with the GRAfT and other cohort study experiences [24, 31].

However, in 2024, Sindu et al. used the same commercial testing platforms and %dd-cfDNA thresholds but observed unsatisfactory sensitivity for detecting ACR or respiratory infection [11]. These diverging experiences are potentially valid and highlight the need to better understand the performance of the test in different patient populations. Future studies should also address multiple reported confounders that limit the assay performance [32–34].

Comparing the fidelity of %dd-cfDNA to more established rejection markers, %dd-cfDNA has a significantly higher sensitivity to detect rejection compared to FEV1 changes, 95% vs. 60%, while offering a more detailed injury map than traditional inflammatory markers such as ESR/CRP [23, 35]. Future studies would benefit from comparing the distinguishing performance of dd-cfDNA compared to ESR/CRP as studied within kidney transplantation [36].

#### Heart Transplant

The Stanford GTD published initial proof of concept for use of %dd-cfDNA in heart populations and the first seminal studies in their single center cohort [16]. In 2019, Khush et al. studied 740 heart transplant patients across 26 centers, pairing them with events of biopsy-proven rejection [17]. Using a 0.2% dd-cfDNA threshold, they reported a 97% NPV for detecting AR. Their findings indicated that %dd-cfDNA detected AR across a broad heart transplant population, not just in lung transplants.

Following those seminal studies, Agbor-Enoh et al. ran a prospective cohort study of 171 subjects through the GRAfT cohort [18]. Notably, AR showed higher %dd-cfDNA compared to controls, with elevations detectable 0.5–3.2 months before histopathologic diagnosis of both ACR and AMR via endomyocardial biopsy. A 0.25% threshold yielded a 99% NPV and could have avoided 81% of endomyocardial biopsies over the study period. Since these initial studies, multiple additional cohorts have emerged to validate diagnostic testing thresholds in AR [19, 20, 37]. We summarize seminal studies in Table 1 and the differing diagnostic thresholds for detecting AR in these cohorts across both heart and lung transplantation.

While most heart transplantation societies do not recommend routine AR screening with troponin/BNP/ESR given their low sensitivity, further studies are needed to directly compare these easily available biomarkers with %dd-cfDNA [2]. When compared directly with endomyocardial biopsy and cardiac MRI, %dd-cfDNA shows sensitivity to detect AR as high as 88% which is higher than MRI alone (85% sensitivity) and EMBx (as low as 58% sensitivity depending on technique) [6, 12, 38]. In the GRAfT cohort, EMB was positive in less than 20% of instances with positive %dd-cfDNA. Of note, %dd-cfDNA was shown to notably not distinguish between patients with angiographic cardiac allograft vasculopathy (CAV) post-transplant and those without, highlighting a particular weakness given the high frequency of and mortality associated with CAV [39].

Like with lung transplantation, experiences have been mixed across centers with–highlighting the challenges that still remain with using %dd-cfDNA routinely in heart transplantation. Institutions have reported inconsistent sensitivities for AR across a range of % cutoffs, high rates of non-rejection causes of elevated dd-cfDNA, and even that patients with elevated dd-cfDNA and negative biopsies had worse outcomes, highlighting areas for future studies [19, 40, 41].

### Cell-Free DNA for Risk Stratification

Studies involving the long-term risk stratification ability of cfDNA have primarily focused on lung transplant populations within the GRAfT consortium, with cfDNA demonstrating consistent predictive performance throughout the transplant journey. In the pre-transplant period, Balasubramanian et al. evaluated 186 lung transplant candidates and reported variable n-cfDNA levels that were two-fold higher than those for healthy controls and were correlated with a patient’s Lung Allocation Score as well as other markers of disease severity [42]. Patients with high levels pre-transplant had higher risks of primary graft dysfunction and death post-transplant. The risk was highest in patients with elevated neutrophil-derived n-cfDNA, suggesting a role for pre-transplant n-cfDNA monitoring for risk evaluation and assessment.

High Early Injury after Transplantation (HEIT) also demonstrates predictive value, particularly injury in the early post-transplant period. In 2016, Agbor-Enoh et al. analyzed a cohort of 108 patients and reported variable %dd-cfDNA in the early post-transplant period. Patients with elevated %dd-cfDNA levels (upper tertile) showed higher rates of AMR, CLAD, and death when compared to those in the lower two tertiles [43]. Alnababteh et al. published a follow up study of rd-cfDNA in 215 patients and found that patients in the upper tertile had lower lung function post-transplant and an increased risk of death and AR when compared to the lower two tertiles [44]. Along a similar vein, Keller et al. evaluated the prognostic role of extreme molecular injury (EMI - measured as %dd-cfDNA above 5%) and found that all episodes of EMI were associated with an increased risk of severe CLAD or death [45]. Put together, there appears to be a close interplay between the allograft and the host which sets the stage for subsequent allograft function, rejection, and other poor outcomes.

Beyond the early post-transplant period, %dd-cfDNA drawn at the diagnosis of multiple acute post-transplant complications has predictive utility. In patients with respiratory pathogens, Bazemore K et al. showed that patients with %dd-cfDNA levels of 1% or higher showed increased risk of CLAD and death [32]. Keller et al. reported that patients with values above 1% at diagnosis of ACR demonstrated increased risk of CLAD and death [46, 47]. %dd-cfDNA levels at the diagnosis of organizing pneumonia and other acute complications are also predictive of CLAD and early death [48].

### Cell-Free DNA to Monitor Infection

Microbial cfDNA (mcfDNA) is found alongside human cfDNA in peripheral blood at lower concentrations and metagenomic sequencing of mcfDNA is an emerging tool that enables unbiased pathogen detection. Currently, there is a commercial clinical-grade mcfDNA sequencing test called the Karius Test that identifies over 1,250 clinically relevant bacteria, DNA viruses, fungi, and parasites non-invasively [49]. Studies have leveraged microbial cfDNA to detect new pathogens in transplant populations and assess a patient’s immunosuppression status [50, 51]. Although this approach has a limitation in identifying colonization versus active infection, it has the potential to detect unculturable and emerging microbes as well as help to distinguish AR from active infection–a known limitation of %dd-cfDNA.

### Monitoring Immunosuppression

De Vlaminck et al. demonstrated a close association between plasma cfDNA and a patient’s degree of immunosuppression post-transplant using plasma anellovirus abundance as a surrogate marker of immunosuppression [50]. Adequate immunosuppression is poised to reduce allograft injury and the risk of AR. Thus, %dd-cfDNA could theoretically assist clinicians in understanding the relative degree of immunosuppression when interpreted alongside traditional laboratory markers. Charya et al. recently tested this hypothesis in the GRAfT cohort. They showed a significant inverse correlation of %dd-cfDNA with both tacrolimus trough concentrations and anellovirus abundance, a recognized surrogate marker of global immunosuppression over time [52]. Percent dd-cfDNA identified episodes of inadequate immunosuppression with higher performance compared to both tacrolimus troughs and anellovirus abundance.

## Adoption of %dd-cfDNA in Routine Clinical Practice

Three CLIA-approved centralized commercial dd-cfDNA tests are available in the US and Europe: AlloSure (CareDx), Prospera (Natera), and TRAC (Eurofins Viracor) [20, 41]. These tests perform %dd-cfDNA testing without the need for prior genotyping. While these assays show considerable agreement in detecting rejection, the cutoff values are different [53]. CareDx also markets a more decentralized testing kit (CE-IVDD) that utilizes custom SNP panels and PCR. Direct comparison of CE-IVDD with a a centralized assay assay demonstrated positive correlation and reproducibility [10]. However, CE-IVDD has a higher assay detection limit, which could reduce its sensitivity, particularly for heart transplants where lower threshold values are needed for diagnosis of AR.

Clinical adopters of %dd-cfDNA often follow variable monitoring protocols given the lack of consensus standards. A recent editorial summarizes common monitoring protocols used in recent years including the ALARM study, which used %dd-cfDNA thresholds of 0.5% and 1% [24, 54]. They found that %dd-cfDNA values above 1% were highly suggestive of AR and served as an “alarm signal”, or a trigger to biopsy and perform additional testing to identify a cause of the derangement. On the other hand, values below 0.5% provide reassurance as an “all clear” signal. Values between 0.5% and 1% represent a gray zone and could serve as an indication for careful monitoring to detect early or impending forms of complications. In light of these results, a recent meta-analysis showed consistency upon review, giving users more guidance on application of %dd-cfDNA although further studies to describe optimal testing windows are still needed [55].

## Gaps in Knowledge

Despite the robust performance of %dd-cfDNA in cohort studies, reports from routine clinical practice show conflicting %dd-cfDNA results, which suggest unaddressed gaps [20, 41, 56]. For example, while replicate analysis demonstrates reproducibility across laboratories, technicians, and platforms, %dd-cfDNA unfortunately presumes stable rd-cfDNA levels post-transplant, which is its first blind spot [57]. This blind spot is particularly problematic, as rd-cfDNA levels can surge and show variable levels after transplantation [21, 58–60]. Any variability therefore results in false-negative or false-positive %dd-cfDNA values independent of the state of allograft injury. Some centers have included absolute dd-cfDNA levels in addition to % to minimize this concern. However, there are significant interindividual differences in dd-cfDNA levels, which can limit its utility [18, 29, 31, 61, 62].

Commercial assays use different %dd-cfDNA thresholds for AR detection, making it challenging to compare results across commercial assays - a 2^nd^ blind spot [10, 63]. There are also no internal control standards to enable comparison between commercial tests. These limitations, plus the paucity of consensus clinical guidelines limit uniform %dd-cfDNA adoption across centers, a 3rd blind spot [20, 41]. Therefore, clinicians and scientists are left to determine their own significant %dd-cfDNA cutoffs for research and clinical purposes. Despite a growing body of evidence for use of %dd-cfDNA, there still remains no uniformly accepted decision-making process published to guide clinician use [2, 8, 64]. Clearly, a standardized approach to research methodology and data validation is required to implement dd-cfDNA beyond its current state, highlighting a key next step towards widespread adoption for transplant care.

%dd-cfDNA testing also lacks specificity for AMR and ACR or between AR and infection–a 4th blind spot. This is a critical shortcoming, as the therapeutic approach to manage ACR, AMR, and various infectious processes differs substantially, with delays or misclassification leading to irreversible allograft injury [23]. A future-ready cfDNA platform could overcome this limitation by coupling quantitative measures from multiple cfDNA compartments - including donor-derived, recipient-derived, and the novel cfDNA testing outlined below - with molecular fingerprints of etiology to produce separate probability scores for AMR and ACR compared to active infection.

Given the limitations outlined above, the emerging field of recipient-derived cfDNA offers a particularly promising avenue for a more holistic approach to post-transplant monitoring. This process may elucidate differences of %dd-cfDNA performance between cohorts and provide inferences to personalize test performance. Only a handful of studies have examined this dimension including our own recent work demonstrating that elevated recipient-derived cfDNA in the early post-transplant period is strongly associated with mortality, AR, and impaired lung function–likely reflecting a systemic injury phenotype that influences the host immune response [44, 65]. In the future, integrating donor and recipient-derived cfDNA into a unified graft–host injury map could quantify both local immune assault and broader physiologic stress, identifying patients at the highest risk for complications such as primary graft dysfunction, secondary infections, or chronic allograft dysfunction long before overt clinical decline.

Do we need randomized control trials (RCTs) in the cfDNA space? There is fear that RCTs, given their high cost, difficulty in achieving enrollment and study benchmarks, could divert resources away from other important discoveries. Well-designed cohort studies have often produced reliable clinical data, particularly in rare diseases as transplantation, without the need for RCTs. However, in the case of %dd-cfDNA, mixed clinical experience compels the need for randomized trials to provide guidelines. A proposed study design for such a trial has been proposed but not yet clinically validated [66]. Any future RCT should ideally address the well-characterized blind spots of %dd-cfDNA to guide proper implementation and adoption into routine clinical practice.

## Next Generation cfDNA Approaches Coming to Transplant Medicine

### Cell-free Nucleic Acids

The human body is complex and composed of various cells, tissues, and organ types, each with specialized functions. Single-cell genetic, transcriptomic, and epigenetic profiling have enabled the comprehensive characterization of cell populations in multiple tissue types–including rare cell types–during both physiologic and diseased states. Advances in next-generation sequencing technologies and computational tools have revolutionized the characterization of the genome, epigenome, and transcriptomic profiles of circulating nucleic acids. This allows researchers to better understand different diseases and pathways related to the disease, and aid in establishing diagnostic methods and therapeutic targets. Cell-free DNA carries genetic, epigenetic, and fragmentomic information related to tissues-of-origin and disease biology.

### Cell-Free RNA

Plasma cfRNA opens a window to capture systemic response, systemic injury, and molecular mechanisms [67]. In addition to traditional RNAs, circulating cfRNA consists of a variety of cfRNA molecules such as microRNAs (miRNAs), short noncoding RNAs (sncRNAs), long non-coding RNAs (lncRNAs) and others that regulate gene expression. Recent studies used circulating messenger RNA to identify risk of preeclampsia in pregnant women and phenotype cancer subtypes [68–70]. There are limited studies on the use of cfRNA in transplantation. Previous studies focused on miRNAs and the results are conflicting [71, 72]. Nonetheless, we believe plasma cfRNA characterization may reveal pathological processes in previously inaccessible organs [73, 74].

### DNA Methylation

Cells show unique epi-methylation markers that play vital roles in genetic regulation with patterns that are unique and stable to each cell type [75]. Recent studies have leveraged cell or tissue specific DNA methylomic markers to identify the tissue origin of cfDNA [76–78]. Microarray- or whole-genome bisulfite sequencing (WGBS) based methods have also successfully been used [79, 80]. While microarrays require high sample input and cover a small percentage of the total methylation site of interest, WGBS, after bisulfite conversion, surveys the methylation state of all cytosines residues and is considered the gold standard. Reference-based deconvolution libraries have continued to grow from an atlas of 25 human cells or tissues to more than 39 cell or tissue types in a recent study [79, 80]. Despite these advances, studies exploring differential methylation regions from cfDNA in transplant settings are scarce. Furthermore, transplant patients are exposed to combinations of immunosuppressive drugs daily, which may cause epigenetic changes that may lead to undesirable outcomes. Therefore, cfDNA methylome analysis may be an additional tool to identify genes and pathways related to AR.

### Cell-Free Histone Modifications

Plasma cfDNA from cell nuclei is wrapped around histone proteins H2A, H2B, H3, and H4 to form an octamer protein called nucleosome, a basic unit of chromatin. Epigenetic profiles of histones, such as mono-methylation of lysine 4 at histone H3 (H3K4me1; enhancer region), carry information related to tissues-of-origin and disease biology. Sadeh et al. recently performed cell-free chromatin immunoprecipitation sequencing (cfChIP-seq) targeting H3K4me3 to delineate gene expression patterns and subsequently enabled the identification of unique biological pathways linked to common disease conditions [81]. Similar studies in cancer biology report similar performance [82, 83]. In heart transplantation, cfChIP-seq demonstrated reliable gene expression signals for immunosuppression therapy including those in the calcineurin and mTOR signaling pathway [52]. This technique may hold significant potential to distinguish between ACR and AMR phenotypes and elucidate genes or molecular pathways associated with rejection for potential therapeutic targets.

### Cell-Free DNA Fragmentomics

cfDNA fragmentation is non-random and regulated by chromatin structure and epigenetic modification. Fragmentation patterns also vary by the cfDNA tissue source and could infer disease biology [84]. Various cell death mechanisms–such as apoptosis, necrosis, autophagy, necroptosis, pyroptosis, ferroptosis, and NETosis–as well as active secretion, contribute distinct pools of cfDNA. Nucleosome positioning controls transcription by restricting access to the target DNA region [85]. Recent studies have leveraged nucleosome positioning to identify tissue origin of cfDNA and decipher gene expression profiles in cells contributing to cfDNA pools [86, 87]. Use of similar techniques in transplant patients could offer further insight into the unique transplant genetic environment and gene expression pathways.

### Cell-Free Mitochondrial DNA

Mitochondria are found in varying numbers and shapes within human cells and differ among cell and tissue type, reflecting metabolic and bioenergetic demands. Mitochondrial DNA (mtDNA) is highly susceptible to oxidative damage due to its poor ability to repair its own DNA [88]. Therefore, during cellular injury, mtDNA is released into the extracellular environment as circulating mt-cfDNA and contribute to the circulating cfDNA pool [89]. A number of studies have reported that increased plasma levels of mtcfDNA in transplant settings can serve as markers of mitochondrial or cell damage, as well as predict allograft dysfunction or episodes of rejection [90–93]. Additionally Ma et al.’s showed that both linear and circular mtDNA coexist in the plasma of liver transplant patients, and as such, both may provide different biological information [94]. Studies characterizing the mt-cfDNA fragment size distribution released from the allograft and recipient tissues may provide new disease-related information.

### Future Directions

**FIGURE 2:**
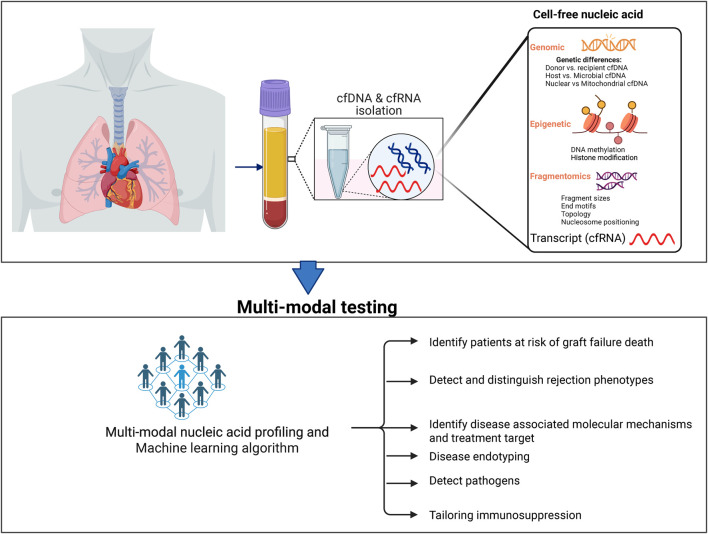
Novel cfDNA and the future diagnostic utility in transplantation.

Figure 2 summarizes the diagnostic potential of %dd-cfDNA and novel technologies discussed. %dd-cfDNA has shown significant results in well-structured cohort studies [18, 43]. However, the mixed results from routine clinical experiences suggest the need for additional studies to address the blind spots and gaps in the dd-cfDNA test [15]. Notably, given the wide variability in host cfDNA levels between patients, it is essential to revisit the effectiveness of %dd-cfDNA across diverse populations, considering personal factors that may impact performance [44]. There is also a need to establish decentralized testing with robust internal controls to enable reproducibility between labs.

Looking ahead, the promise of cfDNA lies in integrating different modalities. Embedding the readouts of these modalities into adaptive algorithms–especially when augmented by pharmacogenomics, immune monitoring, and AI-enabled prediction–could shift practice. For example, this approach could shift immunosuppression from empiric population-based regimens to an individualized, real-time management model. The ultimate vision is a precision-guided strategy where one test informs whether to intensify, taper, or redirect therapy, thereby reducing rejection, infection, and drug toxicity while improving long-term graft survival. While this promise may be a mere dream today, we have summarized novel cfDNA approaches that offer advantages to address these gaps. We anticipate that these new technologies could move transplant monitoring away from the one-size-fits-all paradigm towards a more individualized approach.

## Author Disclaimer

This research was supported in part by the Intramural Research Program of the National Institutes of Health (NIH). The contributions of the NIH authors are considered Works of the United States Government. The findings and conclusions presented in this paper are those of the authors and do not necessarily reflect the views of the NIH or the U.S. Department of Health and Human Services.
